# Artificial Intelligence in the Histopathological Assessment of Non-Neoplastic Skin Disorders: A Narrative Review with Future Perspectives

**DOI:** 10.3390/medsci13020070

**Published:** 2025-06-01

**Authors:** Mario Della Mura, Joana Sorino, Anna Colagrande, Maged Daruish, Giuseppe Ingravallo, Alessandro Massaro, Gerardo Cazzato, Carmelo Lupo, Nadia Casatta, Domenico Ribatti, Angelo Vacca

**Affiliations:** 1 Section of Molecular Pathology, Department of Precision and Regenerative Medicine and Ionian Area (DiMePRe-J), University of Bari “Aldo Moro”, 70124 Bari, Italy; mariodellamura1@gmail.com (M.D.M.); sorino.joana@hsr.it (J.S.); anna.colagrande@gmail.com (A.C.); giuseppe.ingravallo@uniba.it (G.I.); 2 Dorset County Hospital NHS Foundation Trust, Dorchester DT1 2JY, UK; mageddaruish@gmail.com; 3 Department of Engineering, LUM-Libera Università Mediterranea “Giuseppe Degennaro”, S.S. 100-Km-18, Parco il Baricentro, 70010 Bari, Italy; massaro@lum.it; 4 Department of Engineering and Applied Science, University of Bergamo, 24127 Bergamo, Italy; carmelo.lupo@diapath.com; 5 Diapath SpA, 24057 Martinengo, Italy; nadia.casatta@diapath.com; 6 Department of Translational Biomedicine and Neuroscience, University of Bari Medical School, 70121 Bari, Italy; domenico.ribatti@uniba.it; 7 Centro Interdisciplinare Ricerca Telemedicina-CITEL, Università degli Studi di Bari “Aldo Moro”, 70124 Bari, Italy; angelo.vacca@uniba.it

**Keywords:** artificial intelligence, machine learning, deep learning, digital pathology, dermatopathology, skin pathology

## Abstract

Artificial intelligence (AI) is rapidly transforming diagnostic approaches in different fields of medical sciences, demonstrating an emerging potential to revolutionize dermatopathology due to its capacity to process large amounts of data in the shortest possible time, both for diagnosis and research purposes. Different AI models have been applied to neoplastic skin diseases, especially melanoma. However, to date, very few studies have investigated the role of AI in dermatoses. Herein, we provide an overview of the key aspects of AI and its functioning, focusing on medical applications. Then, we summarize all the existing English-language literature about AI applications in the field of non-neoplastic skin diseases: superficial perivascular dermatitis, psoriasis, fungal infections, onychomycosis, immunohistochemical characterization of inflammatory dermatoses, and differential diagnosis between the latter and mycosis fungoides (MF). Finally, we discuss the main challenges related to AI implementation in pathology.

## 1. Introduction

### 1.1. Background and History of Artificial Intelligence

The term artificial intelligence (AI) was coined for the first time in 1956 at the Dartmouth College conference [1], and refers to the use of computer algorithms capable of performing cognitive tasks associated with human intelligence, with the advantage of processing large amounts of data and finding efficient solutions in a shorter time [2]. The first example of AI is recognized to be “El ajedrecista”, created by Torres Quevedo in 1912, representing an embryonal attempt to build a machine capable of playing chess.

### 1.2. Medical Image Recognition in the 1970s

Image recognition was still in its early experimental stages in the 1970s, mostly in academic and defense-related research [2]. The expanding interest in computer vision and artificial intelligence was a major driving force in the discipline and, among the significant advancements and uses, basic pattern recognition and optical character recognition (OCR) were achieved, which are especially useful for handwritten or printed text recognition in banking and postal services (e.g., reading checks); furthermore, to extract features from images, algorithms for edge detection (such as Roberts and Sobel operators) and template matching were created and improved. Governments, particularly the United States (US), have also made investments in computer vision for reconnaissance, target recognition, and satellite picture analysis. Anyway, the majority of image recognition systems were slow and domain-specific due to limitations during the decade, which included low-resolution images, a lack of large training datasets, and insufficient processing power [1,2].

### 1.3. Current Employment

The field of AI is traditionally divided into (1) machine learning (ML), a subset of AI that provides systems with the ability to learn without being explicitly programmed for it, and (2) deep learning (DL), which relies on artificial neural networks (ANNs) organized in multiple layers. DL is a subtype of machine learning, and is particularly adept at tasks involving images, sounds, and language recognition [2]. Moreover, the integration of AI into established fields, such as pathology and dermatopathology, is becoming increasingly common [3,4]. Although AI models have demonstrated proficiency in recognizing lesions like basal cell carcinoma (BCC), seborrheic keratosis (SK), and dermal nevi (DN), further development is needed to improve their ability to differentiate between some challenging melanocytic lesions, such as Spitz nevi, and to diagnose inflammatory conditions [2,5]. With the advent of new technologies and their increasing support in daily diagnostic practice, we believe it is essential for pathologists to have at least a basic understanding of the concepts related to AI, ML, and DL. Furthermore, our aim is to provide an overview of the current state of AI applications in the diagnostic process of non-neoplastic skin diseases, a field that remains to be fully explored.

## 2. Materials and Methods

This review synthesizes the existing English-language literature focusing on the application of AI within the field of non-neoplastic skin pathology, retrieved from a comprehensive search on the PubMed, Web of Science (WoS), and Scopus databases. All articles published on the topic have been evaluated. The keywords of “Artificial Intelligence” and/or “AI” and “Inflammatory dermatopathology” or “non-neoplastic dermatopathology” were used. The last search was performed on 17 January 2025.

## 3. Results

### 3.1. Fundamentals of Artificial Intelligence, Machine Learning, and Deep Learning

#### 3.1.1. Artificial Intelligence

The common denominator underlying both machines and human beings is the ability to process data (i.e., images, sounds) and make decisions. This data-driven decision-making is at the heart of AI [5,6]. Indeed, AI is a rapidly evolving field within computer science that seeks to replicate human intelligence in machines. This involves developing algorithms and systems capable of learning, reasoning, problem-solving, and decision-making, often through the analysis of vast amounts of data. AI encompasses a broad range of techniques, including machine learning and deep learning [1,2,5,6,7].

#### 3.1.2. Machine Learning

ML is a subset of AI that involves the development of algorithms that enable computers to learn from data without explicit programming, using algorithms and statistical models to analyze and draw inferences from patterns in data [8]. This type of learning can be classified as supervised, unsupervised, semi-supervised, or reinforcement learning.

In supervised learning, models are trained using algorithms and datasets where the correct answers are already provided. For example, after being exposed to a large dataset of images labeled as “apple” or “not apple”, a model can learn to discriminate between these two classes based on visual characteristics such as color, shape, and texture [8]. On the other hand, unsupervised learning explores unlabeled datasets to find hidden patterns, discerning and grouping similar data points based on their intrinsic features; for example, given a dataset of various fruits without any prior classification, an unsupervised learning algorithm could cluster together images of apples due to their shared visual characteristics [7,8]. Semi-supervised learning combines the benefits of both supervised and unsupervised learning, utilizing a smaller amount of labeled data in conjunction with a larger amount of unlabeled data to improve model performance, saving time. Reinforcement learning trains agents such as robots or programs to make decisions by interacting with the environment, maximizing a reward signal by selecting actions that lead to favorable outcomes. For instance, a robot learning to open doors could be rewarded when it succeeds, or be penalized for failure [6,9,10].

#### 3.1.3. Deep Learning and Neural Networks

DL derives from ML and is a broad term encompassing a variety of algorithms that are unified by their reliance on ANNs, which are based on the structure of the human brain and can discern intricate patterns within data. ANNs comprise interconnected nodes, or “neurons”, organized into layers, called input, hidden, and output layers. The input layer receives the input data. Then, the latter are propagated through multiple hidden layers, each composed of interconnected artificial neurons, which process and transform the data into a more abstract representation. The final output layer produces the prediction or decision [7,10,11]. In more detail, the hidden layers are instrumental in enabling the network to extract complex features from the data; as a consequence, by increasing the depth of the network (i.e., the number of hidden layers), the model’s capacity to capture intricate relationships and produce accurate predictions is significantly enhanced.

Convolutional neural networks (CNNs), a specialized variant of ANNs, have emerged as the leading technology for medical image analysis, being particularly adept at tasks involving image and video data. They are made up of three different components: convolutional, pooling, and fully connected layers. The convolutional layer extracts specific features from images using nodes: each one recognizes a single element, such as color, shape, or texture [12]. The pooling layers subsequently downsample the feature maps, reducing computational complexity and enabling the network to handle varying input sizes. Finally, the fully connected layers integrate all processed features to generate the final output, such as image classification or object localization (e.g., type or site of the lesion) [6,8,11,12].

### 3.2. Use of AI in Dermatopathology

DL algorithms have demonstrated significant utility in classifying histopathological images of various lesions. The emergence of digital pathology, enabling the acquisition and permanent storage of high-resolution Whole-Slide Images (WSIs), has significantly facilitated picture classification, optimizing the AI training process. At present, the role of AI in dermatopathology is to assist the pathologist and improve diagnostic accuracy, saving time. While AI algorithms have shown great promise in dermatopathology, the development and refinement of these models require a collaborative effort between computer scientists and medical professionals. Dermatopathologists are essential in leading the development and training of AI algorithms in terms of providing expert knowledge on skin diseases, interpreting algorithm outputs, and ensuring that AI systems align with clinical practice [11,13,14]. To date, most ML methods applied to histopathology have concentrated on skin cancers, in particular melanoma. For example, Hekler et al. [15] compared the performance of CNNs in classifying 695 melanocytic neoplasms into nevus or melanoma to that of an expert dermatopathologist, and the AI system demonstrated superior diagnostic accuracy. Indeed, AI is well trained in recognizing melanoma because it relies on a binary classification; it is only able to differentiate between positive and negative images. The diagnosis of non-melanoma skin cancers (NMSCs) is more difficult due to their complex classification and wider range of differential diagnoses, varying from benign to malignant diseases. On the other hand, the field of inflammatory dermatopathology is largely unexplored by AI systems [11,16,17]. To date, AI in non-neoplastic skin diseases has been applied to the following: superficial perivascular dermatitis and their subclassification; psoriasis; fungal infections; onychomycosis; immunohistochemical characterization of inflammatory skin disease; differential diagnosis between benign inflammatory dermatoses (BIDs) and mycosis fungoides (MF).

#### 3.2.1. Superficial Perivascular Dermatitis and Its Subtypes

Superficial perivascular dermatitis represents one of the most encountered types of dermatosis. It can be isolated, as the only histopathological finding (simplex subtype), or associated with other patterns (interface, psoriasiform, or spongiotic subtypes). In this regard, Bao et al. developed an AI-based model to classify subtypes of superficial perivascular dermatitis, selecting 412 cases of interface, spongiotic, and psoriasiform superficial perivascular dermatitis with an established clinicopathological diagnosis and 85 normal skin samples; the simplex subtype was not included because of the scarcity of characterizing pathological elements and the few morphological differences with normal skin. Then, they employed a structure–pattern analysis method, consisting of labeling the pathological features contributing to subtype classifications: 13 features were chosen to this aim, represented by hyperkeratosis, hypergranulosis, acanthosis, liquefaction degeneration of basal cells, lichenoid infiltration, melanophages, spongiosis, inflammatory cell infiltration, blister, parakeratosis, hypogranulosis, thinning of the suprapapillary epidermis, and angiectasia of the dermal papillae. Consequently, WSIs of selected cases were introduced into the AI system as inputs and processed, first employing a DL segmentation model able to recognize the pathological features listed above, and subsequently a DL classification model able to provide an output consisting of the final classification of the image as having a normal, interface, spongiotic, or psoriasiform pattern. Altogether, the accuracy of subtype classification was 85.24%, with a sensitivity of 67.46% and a specificity of 89.09%. In particular, the accuracy was very high for normal skin (>99%), but also for interface dermatitis (83.69%) and spongiotic dermatitis (81.12%); in fact, AI accuracy was the best in recognizing acanthosis, lichenoid infiltration, hypergranulosis, hyperkeratosis and, to a lesser extent, inflammatory cell infiltration, i.e., the key histologic features of interface and spongiotic patterns. The accuracy for the psoriasiform pattern was instead 64.58%. Overall, the results obtained are very close to the actual diagnostic accuracy of dermatopathologists in clinical practice, representing an encouraging preliminary result in this field [18].

#### 3.2.2. Psoriasis

**Figure 1:**
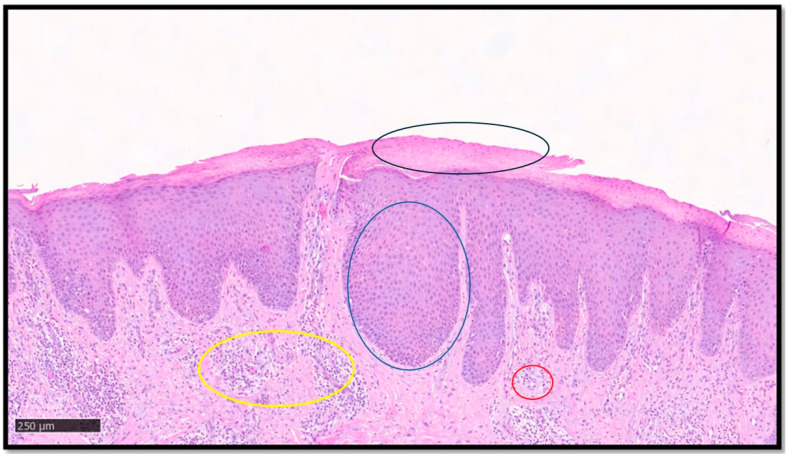
A classic example of psoriasis, characterized by parakeratoses (example in black circle), regular acanthosis (example in blue circle), congested capillaries in the papillary dermis (example in yellow circle), and mixed inflammatory infiltrate with perivascular accentuation (example in red circle). Segmentation of the image, key diagnostic element recognition and interpretation, and final diagnostic output is provided by DL models.

Psoriasis is a chronic immune-mediated inflammatory skin disease affecting approximately 2% of the world population, with high health burden and psychosocial morbidity [19,20,21]. The classic histopathology of psoriasis, when present, is not difficult to identify. Typical cases show parakeratoses with associated hypogranulosis, regular acanthosis with drop-like rete ridges, thinning of the suprapapillary epidermis, congested capillaries in the papillary dermis, and a variable number of neutrophils that may form microabscesses in the stratum corneum or upper epidermis [21]. However, the diagnosis of psoriasis is not always straightforward, because the above features may not necessarily be seen simultaneously and, furthermore, the histological picture may be influenced by several factors, such as the stage of the lesion sampled, the anatomical site, and the clinicopathological subtype [21]. While these difficulties may warrant the need to develop efficient and accurate diagnostic AI models for psoriasis [14], inter-observer subjectivity would present a challenge in providing the ground truth for AI training. Additionally, the current binary nature of most AI algorithms would be a limitation in differentiating psoriasis from potential mimickers, such as seborrheic dermatitis, pityriasis rubra pilaris (PRP), and chronic eczema, among others [21,22]. A first essential step for DL models in recognizing psoriasis histologically, and indeed other inflammatory skin conditions, is to be able to segment the epidermis and dermis, overcoming hurdles such as uneven staining and complex cellularity. This was achieved by Pal et al. [17], who developed a U-shaped CNN using a dataset of 90 psoriasis biopsy pictures: the CNN model successfully analyzed super pixels and classified the skin layers, outperforming other hand-crafted feature-based classifying models. Figure 1 shows an example of psoriasis.

#### 3.2.3. Cutaneous Fungal Infections

**Figure 2:**
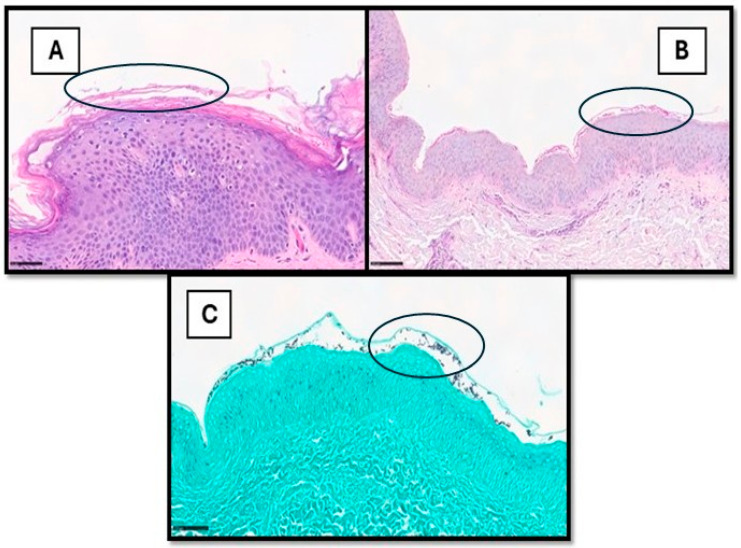
(**A**) This picture shows a histological example of cutaneous superficial fungal infection, in which hyphae and spores can also be readily observed within the stratum corneum via H&E (example in black circle). In other cases (**B**,**C**), they are more subtle and recognized only after careful examination on PAS- or GMS-stained samples (examples in black circles).

Cutaneous fungal infections can be divided into superficial and deep infections, the latter through direct inoculation or spread from other organs [22,23]. The diagnosis of cutaneous fungal infections can be notoriously time-consuming, especially in cases with sparse fungal elements. These cases usually require careful histological examination and the use of special stains, such as periodic acid–Schiff (PAS) and Gomori methenamine silver (GMS) [24,25,26]. Hence, there is potential for using AI models for the detection of fungal organisms, as already reported in other fields of histopathology [27,28,29,30,31]. To date, only a single recent study has investigated the use of DL in identifying fungal organisms in skin tissue sections [26]. The authors used DeePatholgy STUDIO, a do-it-yourself software that allows for the morphological analysis of WSI, bypassing the need to design complex algorithms and the requirement for previous programming knowledge [26]. Tiles with or without fungal elements were annotated on 7 PAS slides (cohort 1) and 15 GMS slides (cohort 2), with the subsequent development of algorithms for each cohort with separate training and validation datasets [26]. The algorithm demonstrated overall low false-positive and false-negative results. Sensitivity, specificity, and F1 score (indicating precision) were higher for GMS (0.93, 0.99, and 0.95, respectively) compared to PAS (0.8, 0.97, and 0.78, respectively) [26]. There are a few limitations in this study, including inherent AI drawbacks, such as the need for ground truth (which can be subjective) and the impact of varying image quality. Additionally, the dependence on special stains meant there was less potential of shortening turnaround times and reducing the use of resources [26,27,28,29,30,31,32]. Figure 2 shows an example of superficial mycosis.

#### 3.2.4. Onychomycosis

Onychomycosis is a common nail infection, and the histological evaluation of nail clippings still appears to be a very sensitive method for diagnosis. Due to its frequency in dermatological practice and the clinical difficulty to differentiate it from other nail diseases, such as nail psoriasis or nail involvement in lichen planus, there is an increased workload for pathologists, resulting in a significant loss of time and resources. To address the diagnostic challenges posed by onychomycosis, Decroos et al. proposed an autonomous AI-based approach for the detection of fungal hyphae in PAS-stained WSIs of nail clippings. This study aimed to reduce the burden of microscopic examination, particularly in specimens with low numbers of micro-organisms. The results showed that AI was non-inferior to four dermatopathologists (with different levels of experience) with regard to specificity and accuracy, but more uncertain in regard to sensitivity. Furthermore, this AI model could offer a promising approach for the preliminary screening of onychomycosis, highlighting regions of interest on WSIs for further evaluation and confirmation by dermatopathologists [33,34].

#### 3.2.5. Immunohistochemical Characterization of Inflammatory Skin Disease

Ding et al. developed an AI-assisted analysis pipeline for immunohistochemical evaluation in inflammatory skin disease. The expression of claudin, cyclin B1, cyclin D1, filaggrin, phospho-histone H2AX, kallikrein 7, Ki67, loricrin, NFAT5, and periplakin was assessed on punch biopsies obtained from eight psoriasis and six atopic eczema patients. The authors applied two DL models on the WSIs of the selected cases to obtain the correct segmentation of epidermal and dermal structures, to exclude common artefacts, and to achieve the subsequent quantitative analysis of immunohistochemical signals. The results proved that the AI-assisted model is effective in achieving the aim, and not inferior to traditional evaluation via microscopy. Therefore, it can be applied in research settings to provide a reliable and time-effective immunohistochemical evaluation of inflammatory skin diseases for a deep understanding of dermatitis. Moreover, it could possibly be introduced in clinical practice in the coming future as new biomarkers emerge in clinical applications in the field of precision medicine [35].

#### 3.2.6. BIDs Versus MF

One of the most challenging differential diagnoses in dermatopathology exists between BIDs and early-stage MF due to their shared clinical and histopathological features, resulting in low inter-pathologist agreement and frequent diagnostic delay. To this aim, in 2020, Scheurer et al. published a DL-aided diagnostic tool for discriminating between eczema and MF based on a dataset of 93 patients; however, the classification accuracy showed an almost negligible difference compared with a dummy classifier [36]. Recently, Doeleman et al. trained weakly supervised models for the differentiation of early MF and BIDs on H&E-stained sections. The results are encouraging: the mean slide-level classification accuracy was around 76%, comparable with the performance of expert pathologists [37,38]. However, an AI-based method integrating clinical and immunohistochemical data, which is essential in the differential diagnosis of MF, has not been achieved to date.

## 4. Discussion

AI is rapidly transforming the medical landscape by processing large amounts of data both for diagnosis and research purposes and obtaining accurate results in short times, and has the potential to revolutionize the field of dermatopathology. To date, the role of AI in the diagnosis of non-neoplastic skin diseases has not been fully explored. Different challenges exist in AI implementation for the evaluation of dermatosis. Herein, we summarize the most important limitations and future perspectives in this regard.

### 4.1. AI Applied to Dermatopathology: Limitations

#### 4.1.1. Complexity of Data

First, inflammatory skin disorders are a very wide and heterogenous group of diseases. Significant inter-observer diagnostic variability exists among pathologists, constituting a major confounding factor for the development of reliable AI-based models. To date, training has usually been carried out on exemplary cases in which a clinical–pathological concordant diagnosis has already been established; however, this situation is distant from daily routine practice, thus representing a selection bias in the machine’s instruction. Furthermore, a significant number of rare or not-well-defined entities exist, of which the collection of a sufficient number for AI training is difficult. Consequently, as AI systems continue to evolve, the integration of diverse data sources, including clinical, dermoscopic, and histopathological data, will be essential to unlock AI’s full potential in assisting dermatopathologists.

#### 4.1.2. Size of Data

The massive size and high pixel density of WSIs pose significant technical hurdles. Consequentially, AI equipment has an elevated cost, limiting its availability to laboratories with adequate financial resources. As a result, the widespread implementation of AI technology is hindered.

#### 4.1.3. Reproducibility of Data

The quality of WSIs is a critical determinant of AI model performance. Suboptimal slide preparation, including contamination, section thickness, and staining quality, can introduce significant noise into the data analysis and reduce the accuracy of AI-based diagnoses. The absence of standardized protocols across different laboratories further exacerbates these challenges; in fact, to date, there is not any type of external validation for the proposed AI-based diagnostic systems.

#### 4.1.4. Interpretation of Data

A multitude of legal and ethical concerns require careful consideration: pathologists represent the figure responsible for the final diagnosis, validating or rejecting the result provided by the machine. Overall, it is important to emphasize that AI cannot replace the role of the physician and cannot provide comprehensive, holistic patient care.

### 4.2. AI Applied to Dermatopathology: Looking Ahead to the Future

**Figure 3:**
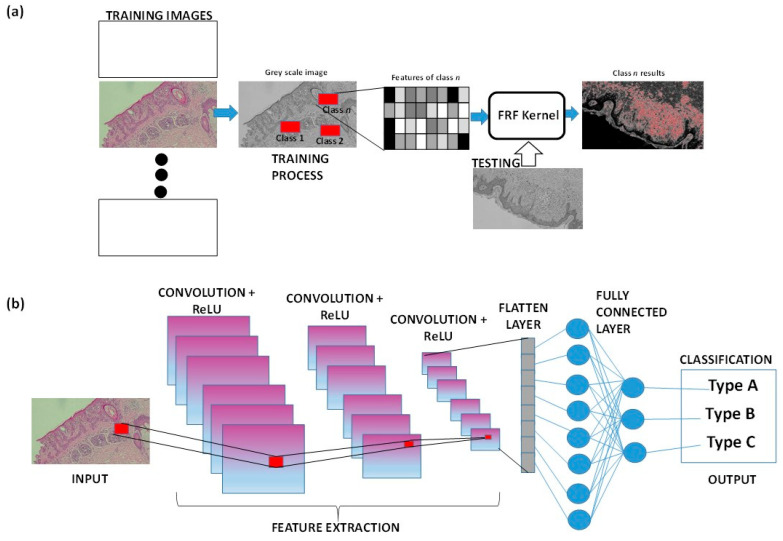
(**a**) FRF algorithm architecture applied to a histological skin image classified as class n. (**b**) Example of CNN architecture classifying a part of the histological image.

As already mentioned, AI-based image tools are suitable to provide new diagnostic procedures. For example, the supervised Fast Random Forest (FRF) algorithm is integrated into clinical protocols and can estimate an alerting threshold [39,40] to discriminate benign from malignant lesions in the analysis of histological images. CNN and Deep Neural Networks are good alternatives for skin disease diagnosis in primary care settings [41]. Fusion image processing techniques can improve the localization and classification of skin lesions by analyzing macro-scale images [42,43], suggesting their potential application for micro- and sub-millimeter-scale images. When focusing on microscopic images, the Reflectance Confocal Microscopy technique can be combined with a CNN to advance non-invasive skin disease diagnosis [44,45]. Figure 3 illustrates a simplified sketch of both approaches, FRF and CNN, in cases of malignant melanoma. For both the approaches, the training images (historical data adopted to construct the training model) are different from the input images (new images to test the extraction of classified features). Once the model has been trained, each image of the historical dataset can be tested again, verifying that it is functioning correctly and identifying sub-areas that were not previously classified (as for the CNN method graph). Convolution involves sliding a filter across the image to create feature maps. ReLU is then applied to the feature maps to keep only the positive values, aiding the CNN in recognizing important patterns in the data (classification output types). The flattening layer is a column-like layer where data (clusters of pixels of features) are converted into a one-dimensional array for feeding the next layer (flatted output of convolutional layer into single long feature vector that is connected to the final classification model, called the fully connected layer) [46].

The Digital Imaging and Communications in Medicine (DICOM) standard [47] could be a reference to define new diagnostic protocols, starting from specific image prerequisites that could modify the classification results. The accuracy of deep learning is confirmed to achieve optimal outcomes in skin disease diagnosis [48], but a lot of work on AI analysis in relation to histopathologic data remains investigational. On the other hand, regulatory agencies in Europe and the United States have approved AI-assisted tools for clinical use in histopathologic diagnosis, including dermatopathology [49].

Important advances also include the application of 3D techniques, such as 3D total body techniques [50,51,52]. Important studies highlight the possibility of combining the processing of histopathology images with sequential dermoscopic ones [53], as well as the possibility of applying tomography and AI to reconstruct 3D epidermal cross-sections to indicate a lesion [54,55,56] or an anomalous vascularization [57,58]. Three-dimensional image processing is also adopted to construct microanatomical structures of skin [59] or 3D lesion models [60], or to perform AI-based virtual staining for non-invasive approaches [61]. Important advances could also be achieved in this regard in the diagnosis of non-neoplastic skin diseases, further applying data fusion techniques with 3D-AI-based image processors.

## 5. Conclusions

AI, particularly its subsets ML and DL, holds great potential for transforming dermatopathology. While AI systems have shown proficiency in diagnosing skin cancers, as well as “simple” non-neoplastic skin diseases, such as fungal infections and onychomycosis, significant challenges remain, including the complexity, size, reproducibility, and interpretation of data. Despite these drawbacks, AI-driven advancements in non-neoplastic skin pathology are promising, particularly in reducing inter-observer variability, optimizing diagnostic accuracy, and saving time. Future developments must integrate diverse data sources, and proper collaboration between dermatopathologists and computer scientists is needed. While AI is not a replacement for human expertise, it can serve as a powerful supplementary tool, highlighting the importance of pathologists gaining foundational knowledge of AI concepts to effectively harness its potential.

## Abbreviations

The following abbreviations are used in this manuscript:

AI	Artificial intelligence
OCR	Optical character recognition
ML	Machine learning
DL	Deep learning
ANN	Artificial neural network
CNN	Convolutional neural network
WSI	Whole-Slide Image
BID	Benign inflammatory dermatosis
MF	Mycosis fungoides
PAS	Periodic acid–Schiff
GMS	Gomori methenamine silver
FRF	Fast Random Forest
